# Therapeutic Effects of Oral Application of Menthol and Extracts from Tormentil (*Potentilla erecta*), Raspberry Leaves (*Rubus idaeus*), and Loosestrife (*Lythrum salicaria*) during Acute Murine Campylobacteriosis

**DOI:** 10.3390/pharmaceutics15102410

**Published:** 2023-10-01

**Authors:** Rasmus Bandick, Lia V. Busmann, Soraya Mousavi, Nizar W. Shayya, Jakub P. Piwowarski, Sebastian Granica, Matthias F. Melzig, Stefan Bereswill, Markus M. Heimesaat

**Affiliations:** 1 Gastrointestinal Microbiology Research Group, Institute of Microbiology, Infectious Diseases and Immunology, Charité-Universitätsmedizin Berlin, Corporate Member of Freie Universität Berlin, Humboldt-Universität zu Berlin, and Berlin Institute of Health, D-12203 Berlin, Germany; rasmus.bandick@charite.de (R.B.); lia.busmann@gmail.com (L.V.B.); soraya.mousavi@charite.de (S.M.); nizar.shayya@charite.de (N.W.S.); stefan.bereswill@charite.de (S.B.); 2 Microbiota Lab, Department of Pharmaceutical Biology, Medical University of Warsaw, 02-097 Warsaw, Poland; jakub.piwowarski@wum.edu.pl (J.P.P.); sebastian.granica@wum.edu.pl (S.G.); 3 Institute of Pharmacy, Freie Universität Berlin, D-14195 Berlin, Germany; matthias.melzig@fu-berlin.de

**Keywords:** tormentil (*Potentilla erecta*), raspberry leaves (*Rubus idaeus*), loosestrife (*Lythrum salicaria*), menthol, plant-derived compounds, *Campylobacter jejuni*, immune modulation, microbiota-depleted IL-10^−/−^ mice, acute campylobacteriosis

## Abstract

Human food-borne infections with the enteropathogen *Campylobacter jejuni* are becoming increasingly prevalent worldwide. Since antibiotics are usually not indicated in campylobacteriosis, alternative treatment regimens are important. We here investigated potential disease-alleviating effects of menthol and of extracts from tormentil, raspberry leaves, and loosestrife in acute murine campylobacteriosis. Therefore, *C. jejuni*-infected microbiota-depleted IL-10^−/−^ mice were orally treated with the compounds alone or all in combination from day 2 until day 6 post-infection. Whereas neither treatment regimen affected gastrointestinal pathogen loads, the combination of compounds alleviated *C. jejuni*-induced diarrheal symptoms in diseased mice on day 6 post-infection. Furthermore, the therapeutic application of tormentil and menthol alone and the combination of the four compounds resulted in lower colonic T cell numbers in infected mice when compared to placebo counterparts. Notably, pro-inflammatory cytokines measured in mesenteric lymph nodes taken from *C. jejuni*-infected mice following tormentil, menthol, and combination treatment did not differ from basal concentrations. However, neither treatment regimen could dampen extra-intestinal immune responses, including systemic pro-inflammatory cytokine secretion on day 6 post-infection. In conclusion, the combination of menthol and of extracts from tormentil, raspberry leaves, and loosestrife constitutes an antibiotic-independent approach to alleviate campylobacteriosis symptoms.

## 1. Introduction

Campylobacteriosis constitutes an infectious enteritis syndrome [1], which represents the most frequent bacterial enteritis of zoonotic origin in the European Union with about 127,000 cases reported in 2020 [2], and furthermore, it impacts global healthcare and economy [3]. The bacterial enteritis syndrome is mainly caused by *Campylobacter jejuni*. The primary reservoirs of the highly motile Gram-negative rod-shaped spiral-curved bacteria are the gastrointestinal tracts of birds and of ruminant mammalian vertebrates [4,5]. *C. jejuni* are transmitted to humans via ingestion of undercooked or even raw contaminated meat, unpasteurized milk or surface waters [4,6]. The clinical manifestation of enteropathogenic infection may range from mild symptoms to acute enterocolitis with abdominal cramps, bloody diarrhea, and fever [1,7]. Treatment strategies include symptomatic measures such as electrolyte substitution, analgesics, or antipyretics, whereas antibiotics are contra-indicated in otherwise healthy individuals. In patients with immune-compromising comorbidities, however, symptoms of acute campylobacteriosis may be more severe due to bacteremia, for instance, and require antibiotic treatment with fluoroquinolones or macrolide antibiotics [8]. Usually, campylobacteriosis symptoms resolve without residues within two weeks post-infection. On rare occasions, however, weeks or even months after recovery from the acute disease, post-infectious intestinal morbidities might occur such as Crohn’s disease, ulcerative colitis or functional dyspepsia. Extra-intestinal sequelae present as Guillain–Barré-syndrome (GBS) or reactive arthritis (RA), for instance [9,10,11]. These autoimmune reactions are caused by antibodies directed against the lipo-oligosaccharides (LOSs) derived from the *Campylobacter* cell wall cross-reacting with distinct sugar molecules of host cell targets [12,13]. Furthermore, both the severity of acute campylobacteriosis and the risk for post-infectious sequelae depend on distinct sialylated LOS structures [14]. The *C. jejuni* LOSs act as endotoxins and activate the innate immune system via Toll-like-receptor-4 (TLR-4) activation and downstream signaling pathways including the mammalian target of rapamycin (mTOR) modulated pathways [15,16]. In turn, apoptotic responses are induced in the intestinal epithelia by the attachment and cellular invasion of *C. jejuni*; inflammation is mainly mediated by interleukin-6 (IL-6), interferon gamma (IFN-γ), and tumor necrosis factor alpha (TNF-α) [17,18].

Since preventive measures including vaccine development failed to guarantee the desired level of population protection against *Campylobacter* infections [19,20], novel therapeutic options become a feasible way to reduce campylobacteriosis cases, with a particular impact on the immune-compromised, including elderly patients or children [8]. As antibiotic therapy is not indicated in the vast majority of cases [21], antibiotic-independent treatment options with immune-modulating compounds constitute promising alternatives. Previous preclinical placebo-controlled studies provided evidence for potent disease-alleviating effects upon oral application of non-toxic plant-derived compounds such as essential oils from clove [22], garlic [23], cumin [24], and cardamom [25], and of polyphenolic compounds including resveratrol [26], and curcumin [27], for instance, in acute murine campylobacteriosis. Hence, traditional phytotherapeutic compounds offer promising therapeutic and preventive options to fight *C. jejuni*-induced disease in humans [28].

Tormentil (*Potentilla erecta* (L.) Raeusch and other *Potentilla* species) has long been used as an herbal remedy for treating diarrheal diseases of different etiology in traditional medicine [29,30]. The disease-alleviating effects of tormentil have been shown to be due to anti-microbial and anti-inflammatory qualities, including the anti-oxidant properties of distinct molecules such as phenolic acids and ellagitannins abundant in the plant [31,32,33].

In herbal medicine, raspberry leaves (*Rubus idaeus* L.) have also been applied for pain relief, and further, for a broad spectrum of indications in obstetrics and dermatology [34,35]. Ellagitannins and phenolic and linoleic acids are the main biologically active compounds in raspberry leaves [36,37], and are responsible for anti-oxidant, anti-inflammatory and anti-microbial effects [38,39,40]. Moreover, in vitro active compounds extracted from raspberry leaves led to relaxation of transmurally stimulated ileum cells isolated from guinea pigs [41]. It is of note that raspberries have been shown to exert bacteriostatic effects against *Salmonella enterica* and *Staphylococcus aureus* through ellagitannins [42]. While extracted phenolic compounds of blackberry and blueberry, both of which belonging to the *Rubus* genus, could inhibit growth of *C. jejuni* in vitro and reduce enteropathogenic adhesion to culture cells [43], ellagitannin-rich raspberry extracts were also shown to exert anti-*Campylobacter* effects in vitro [44].

Purple loosestrife (*Lythrum salicaria* L.) is also known for its anti-oxidant, immune-modulating as well as pain-alleviating effects [45,46], with historic applications in European herbal medicine [47,48]. The main effective compounds in loosestrife are ellagitannins that reduced IL-8 secretion of lipo-polysaccharide (LPS)-stimulated human neutrophils when exposed to loosestrife extracts in vitro. Furthermore, anti-oxidant properties of loosestrife were shown to be due to a reduced release of radical oxygen species [49]. In addition, recent studies revealed antibacterial activities of loosestrife directed against *Staphylococcus aureus*, *Proteus mirabilis*, *Microccocus luteus*, and enteropathogenic *Escherichia coli* [50,51]. Recently, it could be shown that loosestrife administered to ex vivo cultures of piglet gut microbiota was metabolized into active compounds such as urolithins and was paralled by changes in the composition of the gut microbiota [52]. Furthermore, in the case of enteropathogenic *E. coli*, application of loosestrife and ellagitannins resulted in a reduction of cell adhesion in vitro [51].

Also, the phytomedical use of peppermint extracts is well documented [53,54]. The interaction of the peppermint constituent menthol with the thermosensitive transient receptor potential channels has been shown to be responsible for its pain-relieving effects [55,56]. Recent studies further revealed potent anti-microbial properties directed against *S. aureus* and *E. coli*, which also held true for enteropathogens including *Salmonella* Typhimurium and *S. enteritidis* [57,58]. Moreover, menthol application to rats suffering from colitis could ameliorate inflammation [59].

The described immune-modulatory and antibacterial effects of tormentil, raspberry leaves, loosestrife, and menthol prompted us to test the respective compounds alone and all four in combination against acute campylobacteriosis, applying the microbiota-depleted IL-10^−/−^ mouse model. To overcome colonization resistance due to the murine gut microbiota composition preventing mice from stable *C. jejuni* colonization [16,60], conventional IL-10^−/−^ mice were pretreated with broad-spectrum antibiotics [61]. The lack of the *il10* gene renders mice susceptible to the pro-inflammatory effects of the *C. jejuni*-LOS [15]. In consequence, the microbiota-depleted IL-10^−/−^ mice are not only stably infected by the enteropathogen upon oral challenge, but also develop *C. jejuni*-induced acute enterocolitis with bloody diarrhea and wasting symptoms within a week post-infection (p.i.) [16].

In the present study, we perorally treated microbiota-depleted IL-10^−/−^ mice with menthol and the extracts from tormentil, raspberry leaves, and loosestrife from day 2 until 6 following *C. jejuni* infection and monitored the (i) pathogen loads in the gastrointestinal tract and extra-intestinal organs and assessed the (ii) clinical signs as well as (iii) intestinal and (iv) extra-intestinal immune responses during acute campylobacteriosis.

## 2. Materials and Methods

### 2.1. Ethical Statement

The well-being of each mouse was subjected to daily monitoring. The experiments undertaken in the murine infection and inflammation model adhered to the European animal welfare guidelines (2010/63/EU) after receiving approval by the commission for animal experiments (‘Landesamt für Gesundheit und Soziales’, LaGeSo, Berlin, Germany, under the registration number G0104/19). 

### 2.2. Chromatographic Analysis

For chromatographic analysis of the extracts (below), the LC-DAD-IT-MS method was used, applying the Ultimate 3000 series system (Dionex, Idstein, Germany), which was equipped with a diode array detector and linked to an Amazon SL ion trap mass spectrometer (Bruker Daltonik GmbH, Bremen, Germany). Compounds seperation in analyzed extracts was carried out with a Kinetex XB-C18 column (150 mm × 3.0 mm × 2.6 μm), Phenomenex (Torrance, CA, USA). 

The elution process was done with a gradient program as outlined below: The temperature was maintained at 25 °C, starting with 1% B at 0 min and gradually increasing to 26% B over 60 min. The mobile phase flowed at a rate of 0.350 mL/min, with component A being 0.1% formic acid in water and component B being 0.1% formic acid in MeCN (acetonitrile). Each sample, consisting of three microliters, was introduced to the column through the autosampler. UV-visible spectra were recorded within the range of 190 to 800 nm, and chromatograms were acquired at a wavelength of 254 nm. The ion trap Amazon SL mass spectrometer was equipped with an Electrospray Ionization (ESI) interface. The ESI source operated under the following conditions: nebulizer pressure at 40 psi, a dry gas flow rate of 9 l/min, a dry temperature of 134 °C, and a capillary voltage of 4.5 kV. The analysis involved scanning within the m/z range of 70 to 2200. Compounds were analyzed in both negative and positive ion modes, and MS2 analyses were conducted using the Smart Frag mode.

### 2.3. Microbiota Depleted IL-10^−/−^ Mice

IL-10^−/−^ mice (C57BL/6j background) were bred and maintained as reported in detail previously [61]. For the eradication of the commensal intestinal microbiota, three-week-old female and male mice were transferred to sterile cages, and received ampicillin plus sulbactam (2 g/L plus 1 g/L, respectively; Dr. Friedrich Eberth Arzneimittel, Ursensollen, Germany) via the drinking water (ad libitum), lasting for eight weeks, as reported previously [61]. Throughout this period, strict adherence to aseptic conditions was maintained during handling and housing of the mice. Two days before *C. jejuni* infection, the animals transitioned to autoclaved tap water devoid of antibiotics. The complete depletion of the intestinal microbiota in mice was confirmed by both, cultural and molecular analyses of fecal samples, as described earlier [61].

### 2.4. Campylobacter Jejuni Infection

The *C. jejuni* 81-176 bacteria (kept in frozen stocks) were inoculated on selective Karmali agar plates (Oxoid, Wesel, Germany) and incubated for at least 48 h at 37 °C under microaerophilic conditions as described in more detail earlier [16]. Age- and gender-matched microbiota-depleted IL-10^−/−^ mice (3-month-old littermates) underwent peroral infection with 10^9^ colony forming units (CFU) of the pathogen on both day 0 and day 1 by gavage [62].

### 2.5. Compounds and Treatment Regimens

From day 2 until day 6 p.i., mice were treated with the natural compounds via the drinking water (ad libitum). Placebo control mice received autoclaved tap water with sterile phosphate-buffered saline (PBS, Thermo Fisher Scientific, Waltham, MA, USA) ad libitum.

#### 2.5.1. Tormentil

Dried Tormentillae rhizoma were freed from roots of *Potentilla erecta* (L.) Raeusch (Alfred Galke GmbH, Bad Grund, Germany). Then, 100 g of powdered tormentil rootstock were boiled in 1 L of distilled water for 3 min and extracted for another 7 min with stirring. The aqueous extract was filtered after cooling and the filtrate frozen at −20 °C until use and eventually dispersed in autoclaved tap water and sterile PBS to a concentration of 60 mg/L. Regarding the average body weight of 25 g and drinking volume of 5 mL daily, the mice were supplied with approximately 12 mg tormentil per kg body weight per day (Table 1). The chemical composition of the tormentil (TOR) extract assessed by LC-DAD-IT-MS analysis is shown in Supplementary Figure S1 (upper panel) and Supplementary Table S1.

#### 2.5.2. Raspberry Leaves

The dried leaves of raspberries *Rubi idaei folium* Ph.Eur from *Rubus idaeus* L. (Alfred Galke GmbH, Bad Grund, Germany) were powdered and 100 g were extracted with 1 L of boiling distilled water for 10 min with stirring, the aqueous extracts filtered after cooling and frozen at −20 °C. The extract was dispersed in autoclaved tap water and sterile PBS to a concentration of 200 mg/L for use (40 mg per kg body weight per day, Table 1). Results from LC-DAD-IT-MS analysis of the raspberry leaves (RAS) extracts are illustrated in Supplementary Figure S1 (middle panel) and Supplementary Table S1.

#### 2.5.3. Loosestrife

100 g of dried flowering tops of loosestrife *Lythri herba* Ph.Eur of *Lythrum salicaria* L. (Alfred Galke GmbH, Bad Grund, Germany) were powdered and extracted in 1 L of boiling distilled water for 10 min with stirring, and subsequently after cooling, filtered and frozen at −20 °C. The aqueous extract was brought to a concentration of 200 mg/L in autoclaved tap water and sterile PBS for application (40 mg per kg body weight per day, Table 1). Supplementary Figure S1 (lower panel) and Supplementary Table S1 show results from LC-DAD-IT-MS analysis of loosestrife (LOO) extract.

#### 2.5.4. Menthol

Synthetic menthol (Sigma-Aldrich, St. Louis, MO, USA) was dissolved in autoclaved tap water and sterile PBS to a concentration of 500 mg/L (100 mg per kg body weight per day, Table 1). 

### 2.6. Gastrointestinal Pathogen Burdens

Following oral infection, fecal samples were collected daily. Intraluminal gastrointestinal samples were harvested during necropsy (day 6 p.i.). The samples were weighed and homogenized in sterile PBS. The numbers of viable *C. jejuni* bacterial cells were quantified through colony counting after cultivating serial dilutions from the samples on Karmali agar (Oxoid, Wesel, Germany). These were then incubated for at least 48 h at 37 °C under microaerophilic conditions as described previously [16]. The detection limit of viable pathogens was established at 100 CFU per g feces.

### 2.7. Monitoring of Clinical Conditions of Animals

Immediately preceding and on a daily basis after infection, we assessed the clinical conditions of the mice. This was conducted by employing a clinical scoring scheme (maximum 12 points), addressing different clinical indicators. These included symptoms indicative of wasting (scored on a scale of 0 to 4, where 0 is normal; 1 indicates ruffled fur; 2 indicates less locomotion; 3 indicates isolation; and 4 indicates severely compromised locomotion, pre-final aspect), the presence of fecal blood (graded as 0 for absence of blood; 2 for microscopic detection of blood by the Guajac method using Haemoccult, Beckman Coulter/PCD, Krefeld, Germany; and 4 for macroscopic blood visible), and the diarrheal symptoms (assigned scores of 0 for formed feces; 2 for pasty feces; and 4 for liquid feces), as described earlier [18].

### 2.8. Sampling

Blood was obtained by cardiac puncture, and ex vivo biopsies were preserved from the liver, kidneys, mesenteric lymph nodes (MLNs), and colon. Additionally, luminal samples from the stomach, duodenum, ileum, and colon were collected following sacrifice of mice by carbon dioxide asphyxiation on day 6 p.i.

### 2.9. Histopathology

For the histopathological analyses, colonic ex vivo biopsies were immediately fixed in a 5% formalin solution and then embedded in paraffin. Sections (5 µm) were further stained with hematoxylin and eosin (H&E), and subsequently examined by light microscopy (100-times magnification). The assessment of histopathological changes within the large intestines was quantitatively graded according to a histopathological scoring scheme [63]. A score of 0 denoted an absence of mucosal changes and inflammatory cell infiltrates. A score of 1 indicated minimal inflammatory cell infiltrates confined to the mucosa with intact epithelium. A score of 2 depicted mild inflammatory cell infiltrates within both the mucosa and submucosa, coupled with mild hyperplasia and mild goblet cell loss. A score of 3 indicated moderate inflammatory cell infiltrates within the mucosa and submucosa, accompanied by a moderate loss of goblet cells. Finally, a score of 4 indicated a marked inflammatory cell infiltration into both the mucosa and submucosa with marked goblet cell loss, multiple crypt abscesses, and crypt loss [63].

### 2.10. In Situ Immunohistochemistry

Colonic ex vivo biopsies were subjected to fixation using 5% formalin and embedded in paraffin for in situ immunohistochemical analyses, as recently reported [64]. To evaluate apoptotic epithelial cells, neutrophils, T lymphocytes, regulatory T cells, and B lymphocytes, colonic paraffin sections measuring 5 µm were prepared and stained with primary antibodies. The antibodies employed were targeted against specific markers, namely cleaved caspase-3 (Asp175, Cell Signaling, Beverly, MA, USA, 1:200), MPO7 (No. A0398, Dako, Glostrup, Denmark, 1:500), CD3 (no. N1580, Dako, 1:10), FOXP3 (clone FJK-165, no. 14-5773, eBioscience, 1:100), and B220 (no. 14-0452-81, eBioscience; 1:200), respectively. After staining, an independent investigator counted numbers of specifically stained cells from blinded samples using light microscopy. The average number of positively stained cells from each specific stained marker within the blinded samples was determined in six high-power fields (HPF, 0.287 mm^2^, 400-times magnification).

### 2.11. Pro-Inflammatory Cytokines

Intestinal samples were collected from the colon (longitudinally cut specimens of approximately 1 cm^2^, and washed in PBS). Moreover, ex vivo biopsies derived from MLN (3 nodes), liver (1 cm^3^), and kidney (one half after longitudinal cut) were transferred to 24-flat-bottom-well culture plates (Thermo Fisher Scientific, Waltham, MA, USA) containing 500 µL serum-free RPMI 1640 medium (Thermo Fisher Scientific, Waltham, MA, USA) that had been supplemented with both, penicillin (100 µg/mL; 100 µg/mL; Biochrom, Berlin, Germany) and streptomycin (100 µg/mL; Biochrom, Berlin, Germany). The plates were then incubated for 18 h at 37 °C, and organ culture supernatants and serum samples were tested for TNF-α, IFN-γ, and IL-6, by the Mouse Inflammation Cytometric Bead Assay (BD Biosciences, Germany) in a BD FACSCanto II flow cytometer (BD Biosciences). 

### 2.12. Statistics

Data sets were pooled from three independent experiments, and medians and significance levels were computed using GraphPad Prism (version 10.0.1 (218); San Diego, CA, USA). Data were normalized by the Anderson–Darling test. For not-normally-distributed data, multiple comparisons were performed using the Kruskal–Wallis test with Dunn’s post-correction, while for normally distributed data, this was executed using the one-way ANOVA with Tukey post-correction. Two-sided probability (*p*) values ≤ 0.05 were considered significant. 

## 3. Results

### 3.1. Gastrointestinal C. jejuni Numbers Following Oral Treatment of Infected Gut Microbiota-depleted IL-10^−/−^ Mice with Menthol and Extracts Derived from Tormentil, Raspberry Leaves, and Loosestrife Alone or in Combination

**Figure 1:**
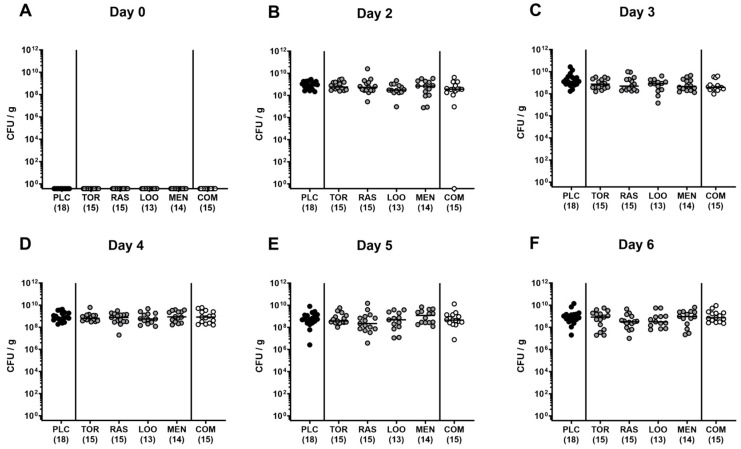
Fecal *C. jejuni* numbers over time following oral treatment of infected gut microbiota-depleted IL-10^−/−^ mice with menthol and extracts derived from tormentil, raspberry leaves, and loosestrife alone or in combination. Mice were orally infected with *C. jejuni* 81-176 strain on days 0 and 1. From day 2 until day 6, mice were treated with either tormentil (TOR), raspberry leaves (RAS), loosestrife (LOO), or menthol (MEN) alone or with a combination of all four compounds (COM) via the drinking water. Placebo control mice received vehicle only (PLC). Pathogen loads were determined in fecal samples collected (**A**) immediately before and (**B**–**F**) at defined time points after infection by culture (in colony-forming units per gram; CFU/g). Medians (black bars) and numbers of mice included from three independent experiments (in parentheses) are indicated.

**Figure 2:**
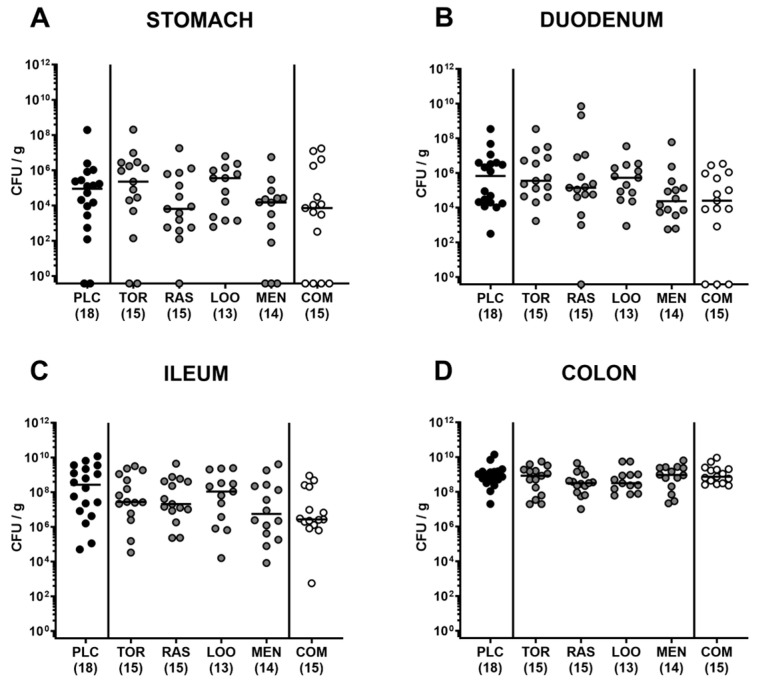
Gastrointestinal *C. jejuni* numbers following oral treatment of infected gut microbiota-depleted IL-10^−/−^ mice with distinct natural compounds alone or in combination. Mice were orally infected with *C. jejuni* 81-176 strain on days 0 and 1. From day 2 until day 6, mice were treated with either tormentil (TOR), raspberry leaves (RAS), loosestrife (LOO), or menthol (MEN) alone or with a combination of all four compounds (COM) via the drinking water. Gastrointestinal pathogen loads were determined in luminal samples collected from the (**A**) stomach, (**B**) duodenum, (**C**) ileum, and (**D**) colon on day 6 post-infection by culture (indicated as colony-forming units per gram; CFU/g). Medians (black bars) and numbers of mice included from three independent experiments (in parentheses) are indicated.

First, we addressed whether treatment of *C. jejuni*-infected microbiota-depleted IL-10^−/−^ mice with either tormentil, raspberry leaves, loosestrife, or menthol alone, or in combination would affect intestinal pathogen loads. Our daily cultural analysis from day 2 until day 6 p.i. revealed no differences in *C. jejuni* numbers within fecal samples taken from the placebo, the single, as well as the combination treatment cohorts (not significant (n.s.); Figure 1). On day 6 p.i., we additionally assessed the *C. jejuni* loads in defined parts of the gastrointestinal tract and determined comparable pathogen counts in the stomach, duodenum, terminal ileum, and colon of mice from all cohorts (n.s.; Figure 2). Hence, treatment with the applied plant-derived compounds alone or in combination did not affect the gastrointestinal colonization capacity of *C. jejuni* in microbiota-depleted IL-10^−/−^ mice.

### 3.2. Clinical Conditions over Time Following Oral Treatment of Infected Gut Microbiota-depleted IL-10^−/−^ Mice with Distinct Natural Compounds Alone or in Combination

**Figure 3:**
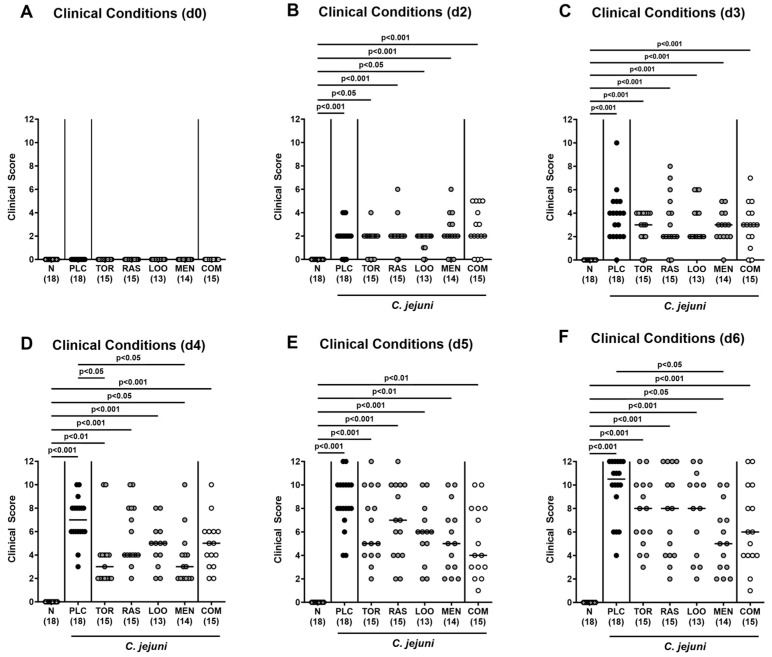
Clinical conditions over time following oral treatment of infected gut microbiota-depleted IL-10^−/−^ mice with distinct natural compounds alone or in combination. Mice were orally infected with *C. jejuni* 81-176 strain on day (d)0 and d1. From d2 until d6, mice were treated with either tormentil (TOR), raspberry leaves (RAS), loosestrife (LOO) or menthol (MEN) alone or with a combination of all four compounds (COM) via the drinking water. Placebo control mice received vehicle only (PLC), whereas naive mice (N) served as uninfected and untreated controls. The clinical conditions of mice were determined (**A**) immediately before and (**B**–**F**) at defined time points after infection by a clinical scoring scheme. Medians (black bars), significance levels (*p* values) determined by the Kruskal–Wallis test with Dunn’s multiple comparison test, and numbers of mice included from three independent experiments (in parentheses) are indicated.

We further assessed the clinical signs of *C. jejuni* infection over time in mice treated with the natural compounds and in placebo-treated control animals. Therefore, we applied a clinical scoring scheme quantitating diarrheal symptoms, abundance of fecal blood, and wasting symptoms. Whereas all mice were clinically uncompromised immediately before infection (Figure 3A), oral *C. jejuni* challenges resulted in increased scores for overall clinical conditions in all groups as early as 24 h after the latest infection (*p* < 0.05–0.001 versus naive; Figure 3B). During the oral treatment period from day 2 until day 6 p.i., elevated clinical scores could be assessed in infected mice irrespective of the treatment regimens (*p* < 0.05–0.001 versus naive; Figure 3C–F). On day 4 p.i., however, lower overall clinical scores were obtained in mice from the tormentil and the menthol treatment cohorts (*p* < 0.05 versus placebo; Figure 3D). At the end of the observation period, mice from the menthol group presented with a better clinical outcome in comparison to placebo control animals, as indicated by lower overall clinical scores in the former versus the latter on day 6 p.i. (*p* < 0.05; Figure 3F). In the case of mice from the combination cohort, at least a trend towards lower overall clinical score could be assessed at the end of the observation period when compared to placebo counterparts (n.s. due to relatively high standard deviations; Figure 3F). 

Furthermore, we analyzed the individual parameters contributing to the total clinical score separately and found that *C. jejuni*-induced increases in scores for fecal blood were comparable in all treatment cohorts (n.s.; Supplementary Figure S2A–C). On day 4 following infection, mice from the placebo, but not the mono-treatment groups, however, exhibited elevated scores quantitating wasting symptoms (Supplementary Figure S2D), whereas at the end of the experiment, *C. jejuni*-induced wasting symptoms were comparably severe in placebo and respective treatment cohorts (n.s.; Supplementary Figure S2F).

**Figure 4:**
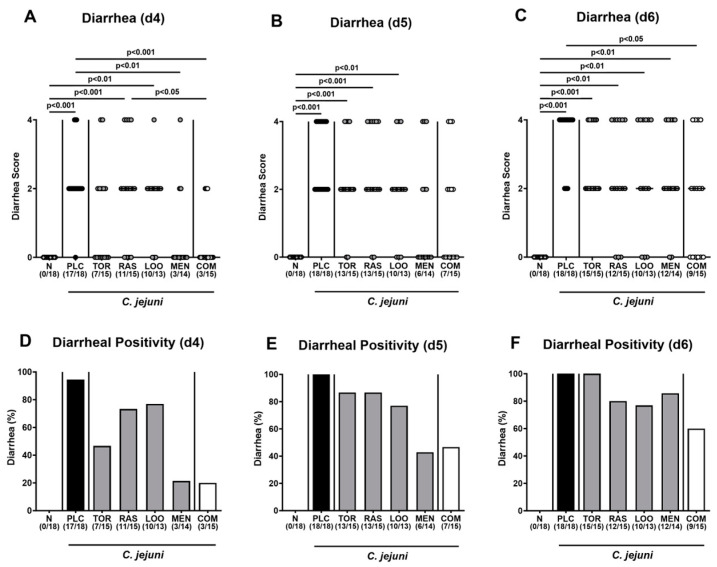
Diarrheal symptoms over time following oral treatment of infected gut microbiota-depleted IL-10^−/−^ mice with distinct natural compounds alone or in combination. Mice were orally infected with *C. jejuni* 81-176 strain on day (d)0 and d1. From d2 until d6, mice were treated with either tormentil (TOR), raspberry leaves (RAS), loosestrife (LOO) or menthol (MEN) alone or with a combination of all four compounds (COM) via the drinking water. Placebo control mice received vehicle only (PLC), whereas naive mice (N) served as uninfected and untreated controls. (**A**–**C**) The severity of diarrheal symptoms was determined at defined time points post-infection by a clinical scoring scheme. (**D**–**F**) The frequencies of diarrheal mice are shown (in %). Medians (black bars), significance levels (*p* values) determined by the Kruskal–Wallis test with Dunn’s multiple comparison test, and numbers of diarrheal mice out of the total number of animals included from three independent experiments (in parentheses) are indicated.

When focusing specifically on the scores assessing the severity of diarrheal symptoms, mice from the combination treatment cohort exhibited lower diarrheal scores on days 4 and 6 p.i. if compared to placebo counterparts (*p* < 0.001 and 0.05, respectively; Figure 4A,C), which also held true for menthol- versus placebo-treated mice on day 4 p.i. (*p* < 0.01; Figure 4A). Notably, diarrheal scores assessed in mice from the menthol group on days 4 and 5 p.i. were similar to those in naive mice (n.s.; Figure 4A,B), whereas the diarrheal scores in the combination cohort and the naive control group were comparable on days 4, 5, and 6 p.i. (n.s.; Figure 4A–C). Furthermore, on day 4 p.i., only 21.4% and 20.0% of mice from the menthol and the combinatory treatment groups, respectively, presented with diarrheal symptoms, whereas this was the case in 94.4% of the placebo control mice (Figure 4D). On days 5 and 6 p.i., all placebo-treated mice were suffering from diarrhea, whereas this held true for 42.9% and 85.7% of the menthol-treated animals and for 46.7% and 60.0% of the mice subjected to the combinatory treatment at respective time points p.i. (Figure 4E,F). Hence, the combined application of all four plant-derived compounds resulted in alleviated pathogen-induced diarrheal symptoms in mice suffering from acute campylobacteriosis.

### 3.3. Inflammatory Signs Following Oral Treatment of Infected Gut Microbiota-Depleted IL-10^−/−^ Mice with Distinct Natural Compounds Alone or in Combination

**Figure 5:**
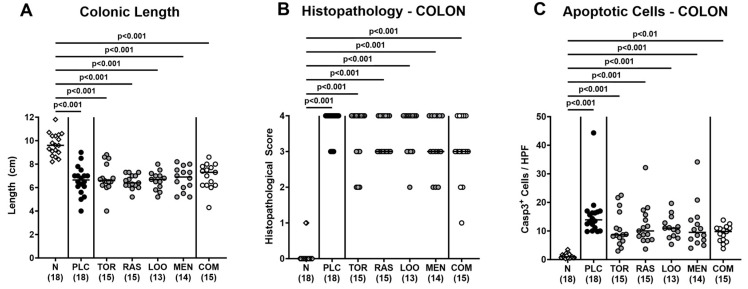
Macroscopic and microscopic inflammatory signs following oral treatment of infected gut microbiota-depleted IL-10^−/−^ mice with distinct natural compounds alone or in combination. Mice were orally infected with *C. jejuni* 81-176 strain on days 0 and 1. From day 2 until day 6, mice were treated with either tormentil (TOR), raspberry leaves (RAS), loosestrife (LOO) or menthol (MEN) alone or with a combination of all four compounds (COM) via the drinking water. Placebo control mice received vehicle only (PLC), whereas naive mice (N) served as uninfected and untreated controls. On day 6 post-infection, (**A**) the colonic lengths were measured (in cm) and (**B**) the histopathological changes in the colon were quantified with a histopathological scoring scheme. Furthermore, (**C**) the apoptotic colonic epithelial cells were counted in paraffin sections of large intestinal explants stained with cleaved caspase-3 (Casp3) and indicated as average numbers out of six representative high-power fields (HPF, 400-times magnification). Medians (black bars), significance levels (*p* values) determined by the Kruskal–Wallis test with Dunn’s multiple comparison test, and numbers of mice included from three independent experiments (in parentheses) are indicated.

We assessed further potential beneficial effects of respective phytomedical treatments on the inflammatory sequelae of *C. jejuni* infection. To address this, we measured the colonic lengths, since intestinal inflammations is known to result in shrinkage of inflamed intestinal compartments [65,66]. In fact, on day 6 p.i., colons of *C. jejuni*-infected mice were shorter as compared to naive mice (*p* < 0.001; Figure 5A) but did not differ between placebo and verum cohorts (n.s.; Figure 5A). Furthermore, the treatment regimens did not affect *C. jejuni*-induced histopathological changes in the colonic mucosa as indicated by comparably elevated histopathological scores in mice treated with the respective compounds alone or in combination (*p* < 0.001 versus naive; n.s. versus placebo; Figure 5B, Supplementary Figure S3A). This was also the case when assessing pathogen-induced colonic apoptosis, given similarly increased numbers of cleaved caspase-3^+^ colonic epithelial cells in verum and placebo-treated mice on day 6 p.i. (*p* < 0.01–0.001 versus naive; n.s. versus placebo; Figure 5C and Supplementary Figure S3B). Of note, the medians of apoptotic colonic epithelial cells were lower in mice following the respective treatment regimens if compared to their placebo counterparts (n.s. due to relatively high standard deviations in the groups; Figure 5C and Supplementary Figure S3B). Hence, the treatment with the plant-derived compounds had no impact on microscopic inflammatory signs of acute campylobacteriosis.

### 3.4. Colonic Immune Cell Responses Following Oral Treatment of Infected Gut Microbiota-Depleted IL-10^−/−^ Mice with Distinct Natural Compounds Alone or in Combination

**Figure 6:**
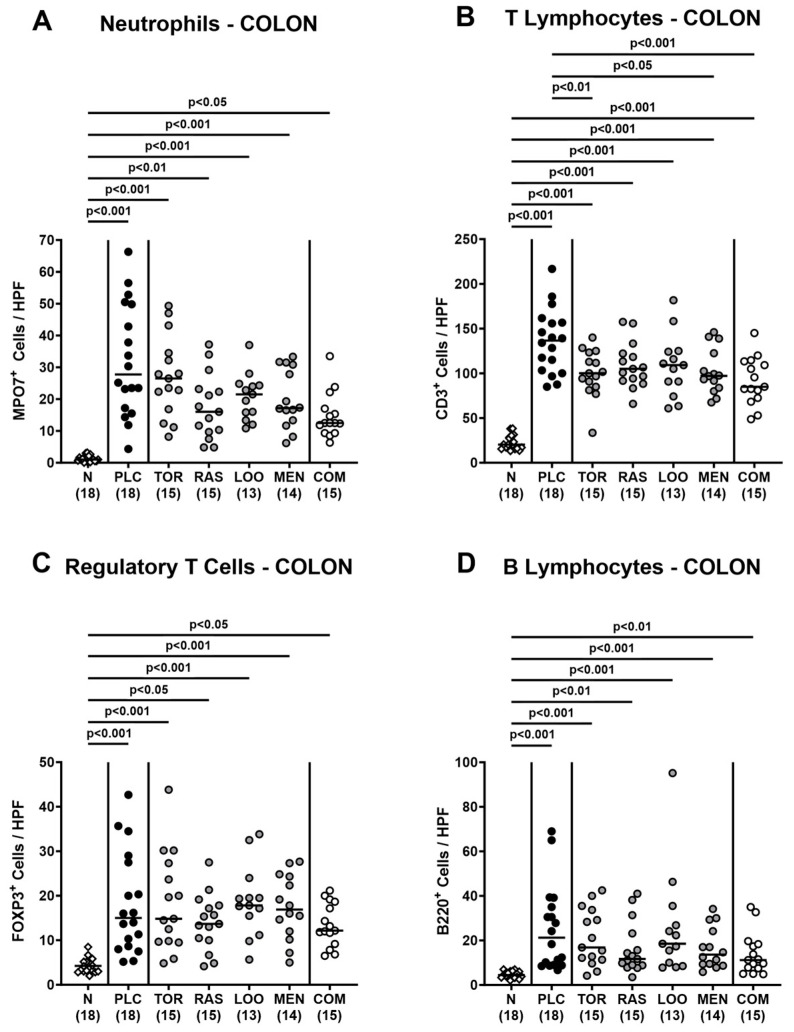
Colonic immune cell responses following oral treatment of infected gut microbiota-depleted IL-10^−/−^ mice with distinct natural compounds alone or in combination. Mice were orally infected with *C. jejuni* 81-176 strain on days 0 and 1. From day 2 until day 6, mice were treated with either tormentil (TOR), raspberry leaves (RAS), loosestrife (LOO), or menthol (MEN) alone or with a combination of all four compounds (COM) via the drinking water. Placebo control mice received vehicle only (PLC), whereas naive mice (N) served as uninfected and untreated controls. On day 6 post-infection, (**A**) neutrophils (MPO7^+^), (**B**) T lymphocytes (CD3^+^), (**C**) regulatory T cells (FOXP3^+^), and (**D**) B lymphocytes (B220^+^) were counted in paraffin sections of large intestinal explants stained with respective antibodies and indicated as average numbers out of six representative high-power fields (HPF, 400-times magnification). Medians (black bars), significance levels (*p* values) determined by the one-way ANOVA test with Tukey’s post-correction (**A**,**B**) and the Kruskal–Wallis test with Dunn’s multiple comparison test (**C**,**D**), and numbers of mice included from three independent experiments (in parentheses) are indicated.

Next, we surveyed potential immune-modulatory effects of the natural compounds in acute campylobacteriosis. Therefore, we stained colonic paraffin sections with antibodies directed against distinct immune cell subsets and enumerated positively stained cells in the colonic mucosa and lamina propria. On day 6 p.i., we detected increased numbers of neutrophils, T lymphocytes, regulatory T cells, and B lymphocytes in all treated groups (*p* < 0.05–0.001 versus naive; Figure 6). The pathogen-induced increases in colonic T lymphocytes were, however, less pronounced in mice treated with tormentil or menthol alone and the combination of all four compounds if compared to placebo counterparts (*p* < 0.05–0.001; Figure 6B).

### 3.5. Intestinal Pro-Inflammatory Cytokine Secretion Following Oral Treatment of Gut Infected Microbiota-Depleted IL-10^−/−^ Mice with Distinct Natural Compounds Alone or in Combination

**Figure 7:**
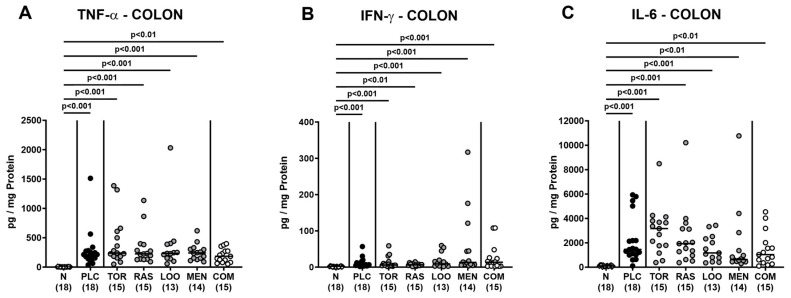
Pro-inflammatory cytokine secretion in the colon following oral treatment of infected gut microbiota-depleted IL-10^−/−^ mice with distinct natural compounds alone or in combination. Mice were orally infected with *C. jejuni* 81-176 strain on days 0 and 1. From day 2 until day 6, mice were treated with either tormentil (TOR), raspberry leaves (RAS), loosestrife (LOO), or menthol (MEN) alone or with a combination of all four compounds (COM) via the drinking water. Placebo control mice received vehicle only (PLC), whereas naive mice (N) served as uninfected and untreated controls. On day 6 post-infection, (**A**) TNF-α, (**B**) IFN-γ, and (**C**) IL-6 concentrations were measured in colonic explants. Medians (black bars), significance levels (*p* values) determined by the Kruskal–Wallis test with Dunn’s multiple comparison test, and numbers of mice included from three independent experiments (in parentheses) are indicated.

**Figure 8:**
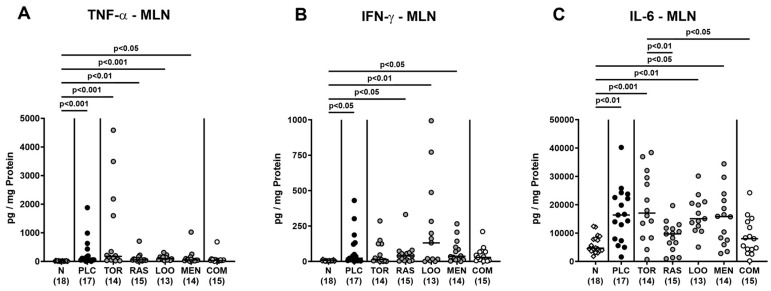
Pro-inflammatory cytokine secretion in mesenteric lymph nodes following oral treatment of infected gut microbiota-depleted IL-10^−/−^ mice with distinct natural compounds alone or in combination. Mice were orally infected with *C. jejuni* 81-176 strain on days 0 and 1. From day 2 until day 6, mice were treated with either tormentil (TOR), raspberry leaves (RAS), loosestrife (LOO), or menthol (MEN) alone or with a combination of all four compounds (COM) via the drinking water. Placebo control mice received vehicle only (PLC), whereas naive mice (N) served as uninfected and untreated controls. On day 6 post-infection, (**A**) TNF-α, (**B**) IFN-γ, and (**C**) IL-6 concentrations were measured in mesenteric lymph nodes (MLN) explants. Medians (black bars), significance levels (*p* values) determined by the Kruskal–Wallis test with Dunn’s multiple comparison test (**A**,**B**), and the one-way ANOVA test with Tukey’s post-correction (**C**), and numbers of mice included from three independent experiments (in parentheses) are indicated.

We next measured pro-inflammatory cytokines secreted in distinct intestinal compartments. *C. jejuni* infection resulted in enhanced secretion of TNF-α, IFN-γ, and IL-6 in colonic ex vivo biopsies (*p* < 0.01–0.001; Figure 7), whereas the cytokine concentrations did not differ between verum- and placebo-treated mice on day 6 p.i. (n.s.; Figure 7). When assessing pro-inflammatory cytokine secretion in MLN draining the inflamed intestines, elevated TNF-α concentrations could be measured in mice from the placebo and the single treatment cohorts on day 6 p.i. (*p* < 0.05–0.001; Figure 8A). Remarkably, infected mice subjected to the combination treatment, however, exhibited naive TNF-α levels in their MLN, which also held true for IFN-γ and IL-6 concentrations, as determined on day 6 p.i. (n.s. versus naive; Figure 8). Furthermore, IFN-γ and IL-6 secretion assessed in MLN taken from tormentil- and raspberry leaves-treated infected mice, respectively, did not exceed basal values (n.s. versus naive; Figure 8B,C). Hence, combinatory treatment of infected mice with the plant-derived compounds dampened pro-inflammatory cytokine secretion in MLN to basal levels.

### 3.6. C. jejuni Translocation to MLN Following Oral Treatment of Infected Gut Microbiota-Depleted IL-10^−/−^ Mice with Distinct Natural Compounds Alone or in Combination

**Figure 9:**
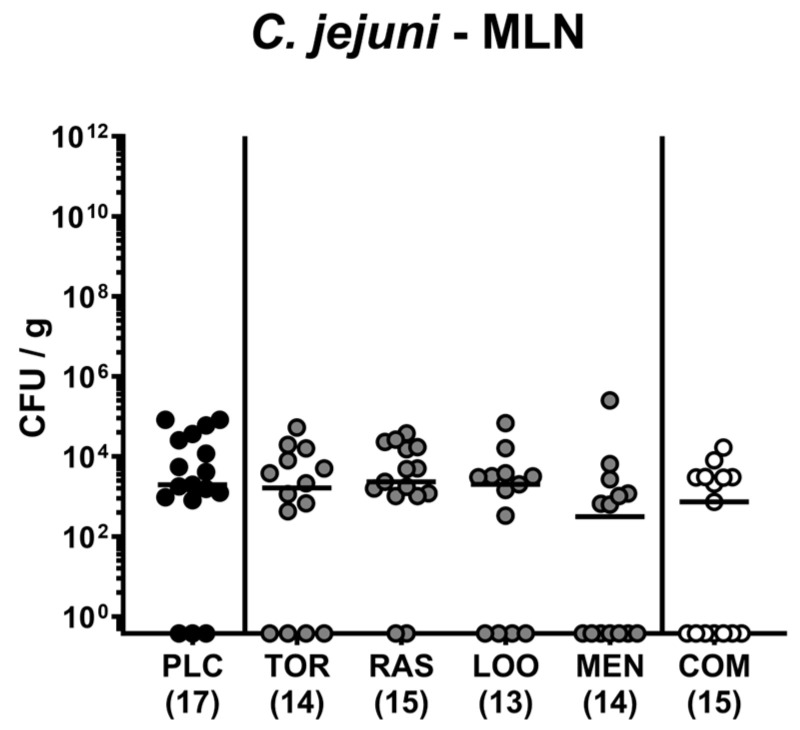
*C. jejuni* translocation to mesenteric lymph nodes following oral treatment of infected gut microbiota-depleted IL-10^−/−^ mice with distinct natural compounds alone or in combination. Mice were orally infected with *C. jejuni* 81-176 strain on days 0 and 1. From day 2 until day 6, mice were treated with either tormentil (TOR), raspberry leaves (RAS), loosestrife (LOO), or menthol (MEN) alone or with a combination of all four compounds (COM) via the drinking water. Placebo control mice received vehicle only (PLC). On day 6 post-infection, *C. jejuni* loads were determined in mesenteric lymph nodes (MLN) by culture. Medians (black bars) and numbers of mice included from three independent experiments (in parentheses) are indicated.

Further, we addressed translocation of live enteropathogens to MLN and found comparable numbers of *C. jejuni* bacteria in homogenates of MLN tissue samples taken on day 6 p.i. (n.s.; Figure 9). Hence, treatment of *C. jejuni*-infected mice with a combination of plant-derived compounds did not reduce the number of enteropathogens that had translocated to the MLN.

### 3.7. Extra-Intestinal Pro-Inflammatory Cytokine Secretion Following Oral Treatment of Infected Gut Microbiota-Depleted IL-10^−/−^ Mice with Distinct Natural Compounds Alone or in Combination

Next, we addressed whether the treatment regimens could alleviate pro-inflammatory cytokine secretion in extra-intestinal organs. *C. jejuni* infection resulted in increases in TNF-α and IFN-γ concentrations in liver, kidneys, and serum and additionally, in elevated renal and serum IL-6 levels measured on day 6 p.i. (*p* < 0.05–0.001; Supplementary Figures S4–S6). These increases in cytokine concentrations occurred, however, independently from the applied treatment regimen (n.s. versus placebo; Supplementary Figures S4–S6). Hence, neither treatment with plant-derived compounds alone nor in combination could alleviate extra-intestinal symptoms including systemic pro-inflammatory cytokine secretion in mice with acute campylobacteriosis. 

## 4. Discussion

Our current placebo-controlled preclinical intervention trial addressed potential anti-pathogenic, disease-alleviating, and immune-modulatory effects of menthol and of extracts derived from tormentil (*Potentilla erecta*), raspberry leaves (*Rubus idaeus*), and loosestrife herb (*Lythrum salicaria*), either alone or in quadruple combination, following oral application in an acute murine campylobacteriosis model. As early as 48 h after initiation of oral tormentil and menthol treatment (i.e., 4 days p.i.), mice presented with less severe clinical signs of campylobacteriosis as indicated by lower overall clinical scores as compared to placebo counterparts (Figure 3D), which also held true for menthol-treated mice at the end of the experiment (Figure 3F). Whereas mice from the placebo, raspberry leaves, and loosestrife groups presented with diarrheal symptoms on day 4 p.i. already, animals that had been subjected to oral tormentil, menthol, and the combination treatment for 2 days only exhibited basal diarrheal scores (Figure 4A). On day 6 p.i., when all placebo control mice were suffering from pronounced diarrhea, 40% of mice from the combination cohort did not present any diarrheal symptoms at all and exhibited diarrheal scores that were comparable to those assessed in naive (i.e., uninfected and untreated) control animals (Figure 4C,F; Table 2). The alleviated clinical, including diarrheal, symptoms observed cannot be explained by relevant anti-pathogenic effects of the treatment regimens, given comparable gastrointestinal *C. jejuni* loads in all infected mice (Figure 1 and Figure 2). 

In support, menthol was previously shown to ameliorate acute colitis symptoms in rats [59] and reduced mortality in lay hens [67]. For peppermint essential oil, of which menthol is a main compound, direct virulence-attenuating effects on *C. jejuni* could be shown [68]. The study revealed a pronounced downregulation of key virulence genes such as *cbf2* and *cadF* mediating cell adhesion, an impaired bacterial motility by disrupting the expression of genes coding for flagellar structures, and even the loss of the characteristic coccoid shape of *C. jejuni* [68]. Furthermore, in a clinical trial, tormentil application improved the clinical conditions in patients suffering from active ulcerative colitis [69]. Notably, in vitro application of loosestrife extracts to IPEC monolayers enhanced the expression of claudin-4 and zona occludens-1, which in turn enhanced cell layer formation and might contribute to the anti-diarrheal effects upon treatment [51].

In our study, the combination of the four distinct compounds as well as menthol and tormentil extract alone reduced the T cell responses in the colon of treated mice in comparison to their placebo counterparts on day 6 p.i. (Figure 6B; Table 2). In support, menthol was shown to reduce lymphocyte differentiation and proliferation [70], whereas extracts derived from *Rubus* species including raspberry could inhibit T lymphocyte activity in vitro [71]. Interestingly, the combined oral application of tormentil (*Potentilla erecta*), raspberry leaves (*Rubus idaeus*), loosestrife herb (*Lythrum salicaria*), and menthol was accompanied by basal TNF-α, IFN-γ, and IL-6 concentrations measured in the MLN on day 6 p.i. (Figure 8; Table 2). Since the numbers of *C. jejuni* that could be isolated from the MLN draining the infected intestines did not differ between the treatment groups (Figure 9), it is highly likely that the basal pro-inflammatory cytokine secretion in the MLN was due to the concerted immune-modulatory actions of the applied compounds. Even upon singular treatment with extracts from tormentil or raspberry leaves, the cytokine concentrations (i.e., IFN-γ and IL-6, respectively) measured in the MLN at day 6 p.i. were comparable to basal levels (Figure 8; Table 2). Interestingly, ellagic acids that are contained in extracts from the *Lythraceae* and *Rosaceae* families, with loosestrife herb and raspberry leaves being respective family members [72], have been shown to decrease expression of pro-inflammatory cytokines such as TNF-α and IL-6 in a TLR-4 dependent manner when applied in murine colitis [73]. As an underlying molecular mechanism, the authors proposed a strongly reduced expression of the mitogen-activated protein kinase (MAPK) and a down-regulation of IL-6 signaling via the signal transducer and activator of the transcription-3 (STAT3) pathway [73]. Likewise, extracts of *Rubus* species resulted in reduced IL-6 expression by inhibition of the STAT3 pathway [71]. In addition, *Potentilla erecta* extracts were shown to down-regulate IL-6 expression in human colonic cells in vitro [32] and to attenuate the nuclear factor ‘kappa-light-chain-enhancer’ of activated B cells (NF-κB)-mediated IL-6-dependent inflammation in stimulated keratinocytes [74]. For menthol, a potent inhibition of LPS-mediated TNF-α and IL-6 secretion could be described that was attributed to adenosine monophosphate-activated protein phosphorylation [75]. Furthermore, menthol was shown to interact with the MAPK and NF-κB pathways and, thereby, protected neurons from experimental LPS-induced Parkinson’s disease [76].

Since *C. jejuni*-LOS constitutes the main enteropathogenic molecule inducing campylobacteriosis upon oral infection [15], it is tempting to speculate that an inhibitory effect of the respective compounds on the LOS-induced TLR-4-mediated pathway was the most likely underlying mechanism for the alleviated diarrheal symptoms during *C. jejuni*-induced enteritis, the dampened colonic T cell responses, and only basal pro-inflammatory cytikine secretion in MLN being observed in combination-treated mice. This hypothesis is supported by results exerted by loosestrife extract in vitro [49], by extracts from *Rubus* species in a murine ulcerative colitis model [77], and by menthol in bovine mammary gland epithelial cells and alveolar macrophages [75,78]. Given the major role of the commensal gut microbiota for the metabolism of extracts from tormentil, loosestrife herb, and raspberry leaves contributing to their anti-inflammatory effects, as reported previously [36], future studies should be performed in a campylobacteriosis model including animals with a defined gut microbiota such as IL-10^−/−^ mice harboring a human gut microbiota, for instance [79].

Our LC-DAD-IT-MS analysis revealed that all three plant extracts contained ellagic acid or its derivates (Supplementary Figure S1, Supplementary Table S1). Ellagic acid and its metabolites, especially urolithins, are known for their potent immune-modulatory (i.e., anti-inflammatory and anti-oxidant) properties [80,81]. Additionally, ellagic acid showed disease-alleviating effects in different in vivo models of colonic inflammation including dextran sulfate sodium (DSS)- and trinitrobenzene sulfonic acid (TNBS)-induced colitis (Table 3) [73,82]. Furthermore, ellagic acid was able to inhibit *Helicobacter pylori* growth in vitro and to ameliorate *H. pylori*-induced murine gastritis in vivo (Table 3) [83]. Whereas the bioavailability of ellagic acid is rather low, its non-adsorbed components are further metabolized to various urolithin derivatives by distinct commensal gut bacterial members of the colonic microbiota [84]. Hence, the depletion of the gut microbiota in antibiotic-pre-treated IL10^−/−^ mice applied here could be one of the reasons for the limited anti-inflammatory effects observed upon ellagic acid treatment in acute murine campylobacteriosis. 

Besides ellagic acid, further biologically active components of the applied compounds such as procyanidin, gallic acid, and agrimoniin (in tormentil), quercetin (in raspberry leafs), and vescalagin, as well as castalagin (in purple loosestrife) were identified (Supplementary Figure S1, Supplementary Table S1), which have already been shown to exert antibacterial and immune-modulaory effects, both in vitro and in vivo [49,85,86,87,88,89,90,91,92,93,94,95,96,97] (Table 3). Interestingly, procyanidin detected in tormentil inhibited the growth of enteropathogens such as *Listeria monocytogenes* and *C. jejuni* in vitro [85,86] and ameliorated DSS-induced colitis in mice [87,88] (Table 3).

**Table 3:** Summary of the known antibacterial and immune-modulatory effects by the applied compounds.

Extract	Compound	Study	Summary of the Effects	Ref.
**TOR** **RAS** **LOO**	**Ellagic acid**	In vivo (mice)	Antibacterial activity against *Helicobacter pylori*	[83]
		TNBS-induced colitis in rats	Downregulated iNOS mRNA (colon) Lower TNF-α concentrations (colon)	[82]
		DSS-induced colitis in mice	Less intestinal inflammation Less diarrhea Attenuated histopathological changes (colon) Downregulated COX-2 and iNOS mRNA (colon) Lower IL-6, IFN-γ, TNF-α concentrations (colon)	[73]
**TOR**	**Procyanidin**	In vitro	Antibacterial activity against *Campylobacter jejuni*	[85]
		In vitro	Antibacterial activity against *Listeria monocytogenes*	[86]
		DSS-induced colitis in mice	Less severe colitis Decreased colonic infiltration with macrophages Downregulated IL-1β, TNF-α, IL-6 mRNA Suppressed NF-κB signaling	[87]
		DSS-induced colitis in mice	Less severe colitis Reduced shortening of colonic length Decreased proinflammatory macrophages Downregulated IL-1β, IL-6, TNF-α, iNOS mRNA Upregulated TGF-β, CD206 mRNA	[88]
	**Gallic acid**	DSS-induced colitis in mice	Reduced shortening of colonic length Enhanced reconstruction of microvilli (colon) Attenuated histopathological changes (colon) Downregulated TNF-α, IL-1β, IFN-γ, IL-6, IL-17 mRNA (colon)	[89]
		TNBS-induced colitis in mice	Attenuated histopathological changes (colon) Inhibited apoptosis (colon) Upregulated IL-4, IL-10 mRNA (colon) Downregulated IL-1, IL-6, IL-12, IL-17, IL-23, TGF-β, TNF-α mRNA (colon) Suppressed NF-κB signaling	[90]
	**Agrimoniin**	In vitro	Antibacterial activity against *Helicobacter pylori*	[96]
		In vitro	Inhibited IL-8 secretion in TNF-α -treated human gastric epithelial cells Suppressed NF-κB signaling	[97]
**RAS**	**Quercetin**	In vitro	Antibacterial activity against *Klebsiella pneumoniae*, *Pseudomonas aeruginosa*, *Yersinia enterocolitica*	[91]
		In vitro	Antibacterial activity against *Vibrio parahaemolyticus*	[92]
		Acetic acid-induced colitis in mice	Attenuated histopathological changes (colon) Decreased recruitment of neutrophils (colon) Lower IL-1β, IL-33 concentrations (colon) Higher IL-10 concentrations (colon)	[93]
		DSS-induced colitis in mice	Less severe colitis Attenuated histopathological changes (colon) Decreased IFN-γ+ and TNF-α+ CD4+ T cells (colon, MLN) Lower TNF-α, IFN-γ, IL-6 concentrations (colon) Upregulated IL-10 mRNA (colon)	[94]
**LOO**	**Vescalagin and ** **castalagin**	In vitro	Antibactericidal activity against *Staphylococcus epidermidis*, *Staphylococcus aureus*, methicillin-resistant *Staphylococcus aureus*, and *Pseudomonas aeruginosa*	[95]
		In vitro	Inhibited IL-8 production from human neutrophils	[49]

TOR: tormentil, RAS: raspberry leaves, LOO: loosestrife; DSS: dextran sulfate sodium, TNBS: Trinitrobenzene sulfonic acid, iNOS: inducible nitric oxide synthase, COX: cyclooxygenase, TGF-β: transforming growth factor beta, MLN: mesenteric lymph nodes.

Treatment of mice suffering from DSS or TNBS colitis with gallic acid, also found in tormentil (Supplementary Figure S1, Supplementary Table S1), resulted in reduced colonic TNF-α and IFN-γ concentrations [89,90] (Table 3), which further supports our results of basal IFN-γ concentration in MLN taken from tormentil-treated *C. jejuni*-infected mice (Table 2).

Moreover, previous studies revealed antibacterial and anti-inflammatory properties of quercetin found in raspberry leaves’ extract. In fact, quercetin application could inhibit the growth of defined Gram-negative bacteria such as *Klebsiella pneumoniae*, *Pseudomonas aeruginosa*, *Yersinia enterocolitica* [91], and *Vibrio parahaemolyticus* [92], for instance. Furthermore, exogenous quercetin suppressed the pro-inflammatory immune responses in murine DSS and acetic acid-induced colitis [93,94] (Table 3), as indicated by inhibited colonic recruitment of neutrophils and T lymphocytes [93,94] and decreased IL-6 concentration in DSS colitis mice [94] (Table 3), further supporting our presented data (Table 2).

In loosestrife, mainly *C*-glucosidic ellagitannins such as vescalagin, castalagin, and dimeric salicarinins A and B were identified (Supplementary Figure S1, Supplementary Table S1). Although distinct antibacterial and anti-inflammatory effects of these ellagitannins have been reported [49,95] (Table 3), the loosestrife treatment of *C. jejuni*-infected IL-10^−/−^ mice had no biologically relevant impact on the disease outcome in our study (Table 2).

## 5. Conclusions

Our preclinical placebo-controlled intervention study provides initial evidence that the combined oral application of menthol and extracts of tormentil (*Potentilla erecta*), raspberry leaves (*Rubus idaeus*), and loosestrife (*Lythrum salicaria*) constitutes a promising antibiotic-independent approach to alleviate *C. jejuni*-induced enteritis. Further non-toxic natural compounds with known anti-inflammatory and/or anti-pathogenic effects should be tested to alleviate or even prevent food-borne enteropathogenic diseases including campylobacteriosis.

## Figures and Tables

**Table 1:** Treatment regimens and concentrations of applied substances.

Treatment	Daily Dose (mg/kg)	Drinking Solution (mg/L)	Minimal Inhibitory Concentration (mg/L)
**Tormentil**	12	60	>3840
**Raspberry leaves**	40	200	>12,800
**Loosestrife**	40	200	>12,800
**Menthol**	100	500	16,000
**Combination**	192	960	>15,360

**Table 2:** Summary of the results.

Treatment	Results
**TOR**MENTIL	- 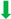 T lymphocytes (colon) vs. PLC - basal IFN-γ (MLN)
**RAS**PBERRY LEAVES	- basal IL-6 (MLN)
**LOO**SESTRIFE	- no significant effect
**MEN**THOL	- improved clinical outcome vs. PLC - 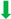 T cells (colon) vs. PLC
**COM**BINATION	- 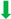 diarrheal symptoms vs. PLC - 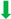 T lymphocytes (colon) vs. PLC - MLN: basal TNF-α basal IFN-γ basal IL-6

PLC: placebo, MLN: mesenteric lymph nodes.

## Data Availability

The data presented in this study are available on request from the corresponding author.

## Supplementary Materials

The following supporting information can be downloaded at: https://www.mdpi.com/article/10.3390/pharmaceutics15102410/s1, Figure S1: LC-DAD-IT-MS analysis of tormentil (TOR), raspberry leaves (RAS), and loosestrife (LOO) extracts. Table S1: Chemical composition of tormentil (TOR), raspberry leaves (RAS), and loosestrife (LOO) extracts determined by the LC-DAD-IT-MS analysis. Figure S2: Abundance of fecal blood and wasting symptoms over time following oral treatment of infected gut microbiota-depleted IL-10^−/−^ mice with distinct natural compounds alone or in combination. Figure S3: Representative photomicrographs illustrating microscopic inflammatory changes following oral treatment of infected gut microbiota-depleted IL-10^−/−^ mice with distinct natural compounds alone or in combination. Figure S4: Pro-inflammatory cytokine secretion in the liver following oral treatment of infected gut microbiota-depleted IL-10^−/−^ mice with distinct natural compounds alone or in combination. Figure S5: Pro-inflammatory cytokine secretion in the kidneys following oral treatment of infected gut microbiota-depleted IL-10^−/−^ mice with distinct natural compounds alone or in combination. Figure S6: Systemic pro-inflammatory cytokine secretion following oral treatment of infected gut microbiota-depleted IL-10^−/−^ mice with distinct natural compounds alone or in combination.
