# Supplementary material for: Therapeutic Effects of Oral Application of Menthol and Extracts from Tormentil (Potentilla erecta), Raspberry Leaves (Rubus idaeus), and Loosestrife (Lythrum salicaria) during Acute Murine Campylobacteriosis
Source: Pharmaceutics. 2023 Oct 1;15(10):2410. doi: 10.3390/pharmaceutics15102410 (PMC10610364; doi:10.3390/pharmaceutics15102410)
Supplement: Supplementary file 1 [file pharmaceutics-15-02410-s001.zip › pharmaceutics-2564473-supplementary.pdf]

Therapeutic effects of oral application of menthol and extracts from tormentil (*Potentilla erecta*), raspberry leaves (*Rubus idaeus*), and loosestrife (*Lythrum salicaria*) during acute murine campylobacteriosis

Rasmus Bandick[1], Lia V. Busmann[1], Soraya Mousavi[1], Nizar W. Shayya[1], Jakub P. Piwowarski[2], Sebastian Granica[2], Matthias F. Melzig[3], Stefan Bereswill[1§], Markus M. Heimesaat[1§#]

[1] Gastrointestinal Microbiology Research Group, Institute of Microbiology, Infectious Diseases and Immunology, Charité - Universitätsmedizin Berlin, corporate member of Freie Universität Berlin, Humboldt-Universität zu Berlin, and Berlin Institute of Health, D-12203 Berlin, Germany

[2] Microbiota Lab, Department of Pharmaceutical Biology, Medical University of Warsaw, 02-097 Warsaw, Poland

[3] Institute of Pharmacy, Freie Universität Berlin, D-14195 Berlin, Germany

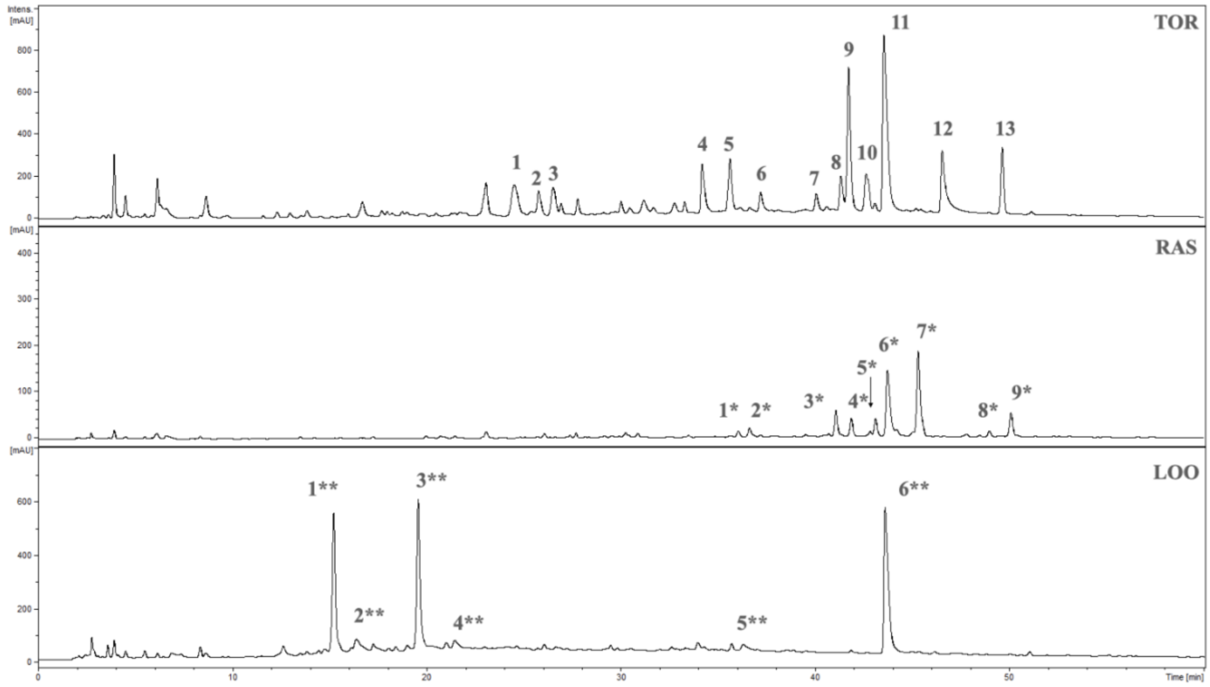

Supplementary Figure S1: LC-DAD-IT-MS analysis of tormentil (TOR), raspberry leaves (RAS), and loosestrife (LOO) extracts**.** Peaks are labelled with the respective compound numbers according to the data displayed in Table S1.

Supplementary Table S1: Retention Times and MS² Data for tormentil (TOR), raspberry leaves (RAS), and loosestrife (LOO) extracts.

Extract	Number	Identification	retention time [min]	λ max (nm)	m/z [M – H]⁻	MS² ions	Reference
TOR	1	dimeric procyanidin B type	24.5	278	**577**	559, 451, **425b**, 407, 289	10.1002/pca.2570
	2	catechin	25.8	278	**289**	245b, 205, 125	10.1002/pca.2570
	3	trimeric procyanidin C type	26.5	277	**865**	847, 739, 695b, 577, 425, 407, 287	10.1002/pca.2570
	4	pentameric procyanidin	34.6	279	**1441**	1153b, 863, 575, 289	10.1002/pca.2570
	5	tertameric procyanidin	36.3	279	**1153**	1027, 983, 865b, 863, 695	10.1002/pca.2570
	6	leavigatin 1, 2 or 3	37.3	268	**1567**	934b, 783, 633	10.1002/pca.2570
	7	gallic acid derivative	40.3	269	**511**	465b, 221	-
	8	leavigatin 1, 2 or 3	41.5	268	**1567**	934b, 783, 633	10.1002/pca.2570
	9	ellagic acid pentoside	41.9	251, 360	**433**	301b	10.1002/pca.2570
	10	methylellagic acid hexoside	42.7	250, 364	**477**	315b	10.1002/pca.2570
	11	ellagic acid	43.9	250, 367	**301**	230b	10.1002/pca.2570
	12	agrimoniin	46.7	268	**1869**	1567b, 1085	10.1002/pca.2570
	13	methylellagic acid pentosdie	49.7	246, 364	**447**	315b, 300	10.1002/pca.2570
RAS	1*	phenolic acid derivative	36.2	255, 300, 324	**339**	321, 177b	-
	2*	sanguiin H-10 isomer	36.8	268	**1568**	1235, 935, 783b, 301	10.3390/plants10112317
	3*	quercetin rhamnohexoside	41.2	255, 352	**609**	301b, 179	10.3390/plants10112317
	4*	quercetin pentoside	42.0	254, 351	**433**	301b	10.3390/plants10112317
	5*	ellagic acid pentoside	43.2	255, 360	**433**	301b, 191	10.3390/plants10112317
	6*	ellagic acid	43.8	252, 367	**301**	255b	10.3390/plants10112317
	7*	quercetin glucuronide	45.6	256, 352	**477**	301b, 227, 151	10.3390/plants10112317
	8*	quercetin derivative	48.6	254, 351	**607**	505, 463b, 301	10.3390/plants10112317
	9*	kaempferol glucuronide	50.2	264, 340	**461**	285b, 257	10.3390/plants10112317
LOO	1**	vescalagin	15.3	260	**933**	915b, 782, 570, 445, 301	10.1002/pca.2415
	2**	salicarinin A	16.5	259	**1867**	1083, 933b, 631, 301	10.1002/pca.2415
	3**	castalagin	19.3	260	**933**	915, 897, 631b, 441	10.1002/pca.2415
	4**	salicarinin B	21.7	260	**1867**	1547, 915b, 631, 301	10.1002/pca.2415
	5**	isoorientin	36.5	262, 351	**447**	429, 357, 327	10.1002/pca.2415
	6**	ellagic acid	43.8	252, 364	**301**	255b, 241	10.1002/pca.2415

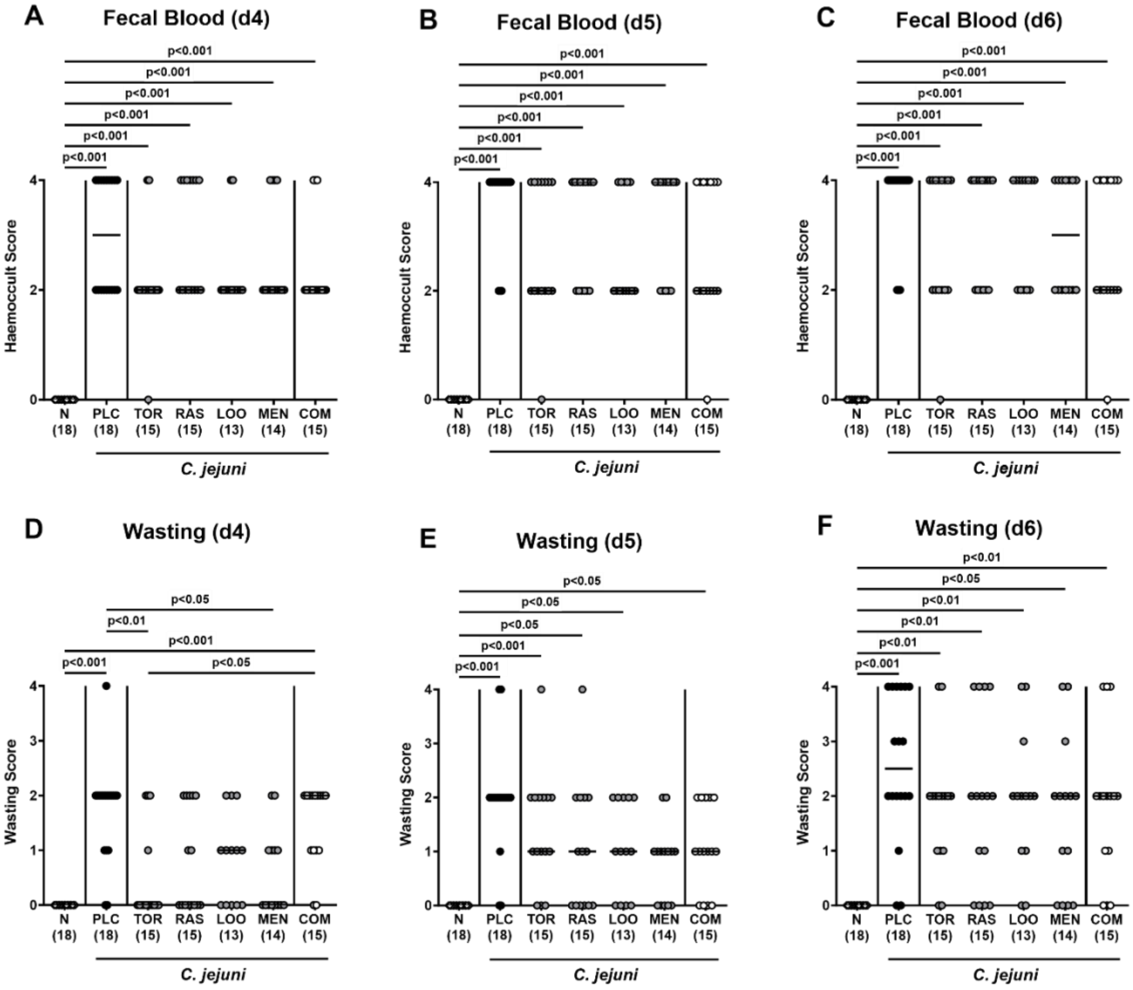

Supplementary Figure S2: Abundance of fecal blood and wasting symptoms over time following oral treatment of infected gut microbiota depleted IL-10$^{-/-}$ mice with distinct natural compounds alone or in combination. Mice were orally infected with *C. jejuni* 81-176 strain on days 0 and 1. From day 2 until day 6, mice were treated with either tormentil (TOR), raspberry leaves (RAS), loosestrife (LOO), and menthol (MEN) alone or with a combination of all four compounds (COM) via the drinking water. Placebo control mice received vehicle only (PLC), whereas naive mice (N) served as uninfected and untreated controls. Severities of clinical campylobacteriosis signs such as **(A-C)** fecal blood and **(D-F)** wasting symptoms were determined at defined time points post-infection by clinical scoring schemes. Medians (black bars), significance levels (p values) determined by the Kruskal-Wallis test with Dunn's multiple comparison test, and numbers of mice included from three independent experiments (in parentheses) are indicated.

A

Histopathology - COLON

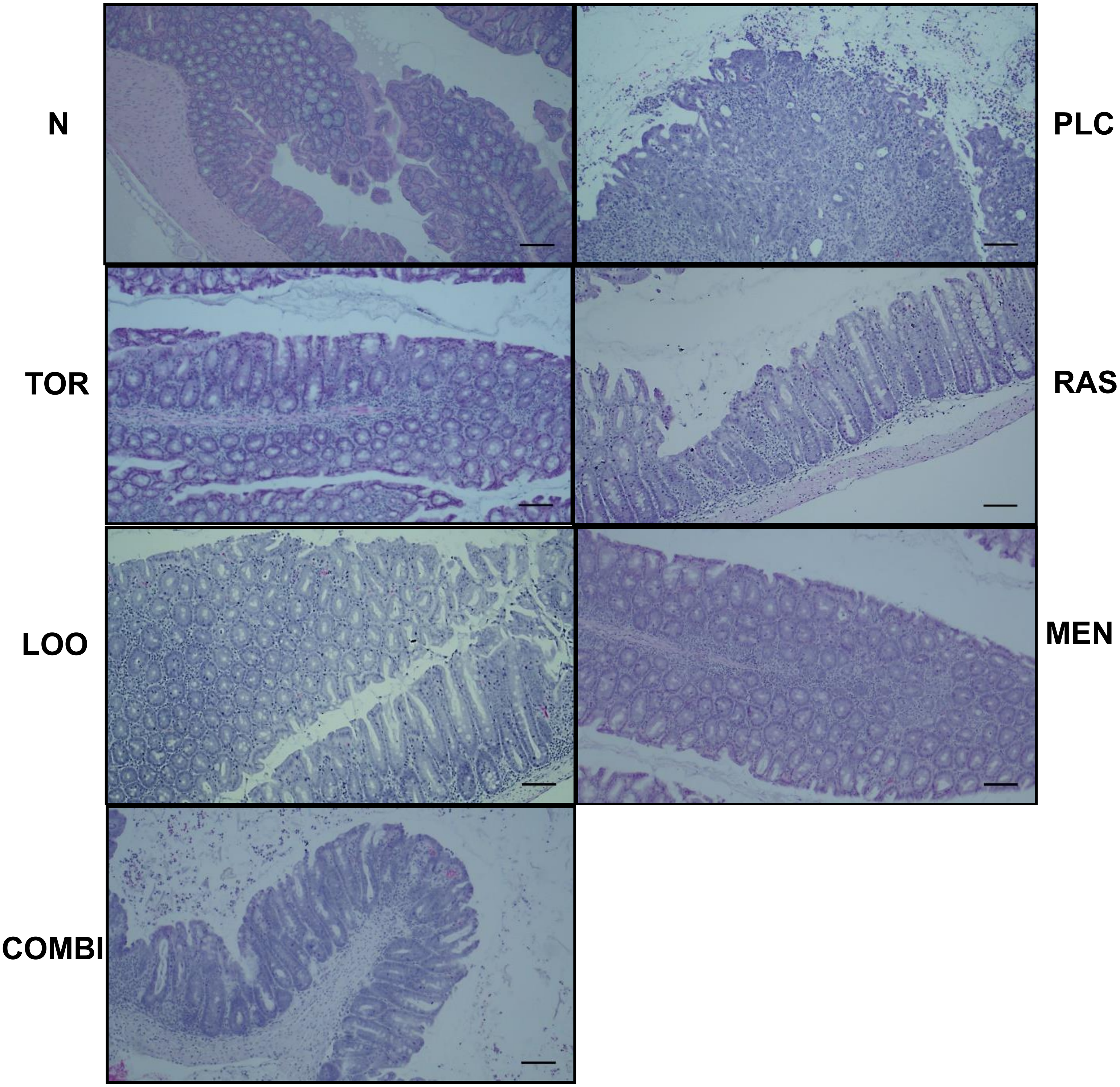

B Apoptotic Cells - COLON

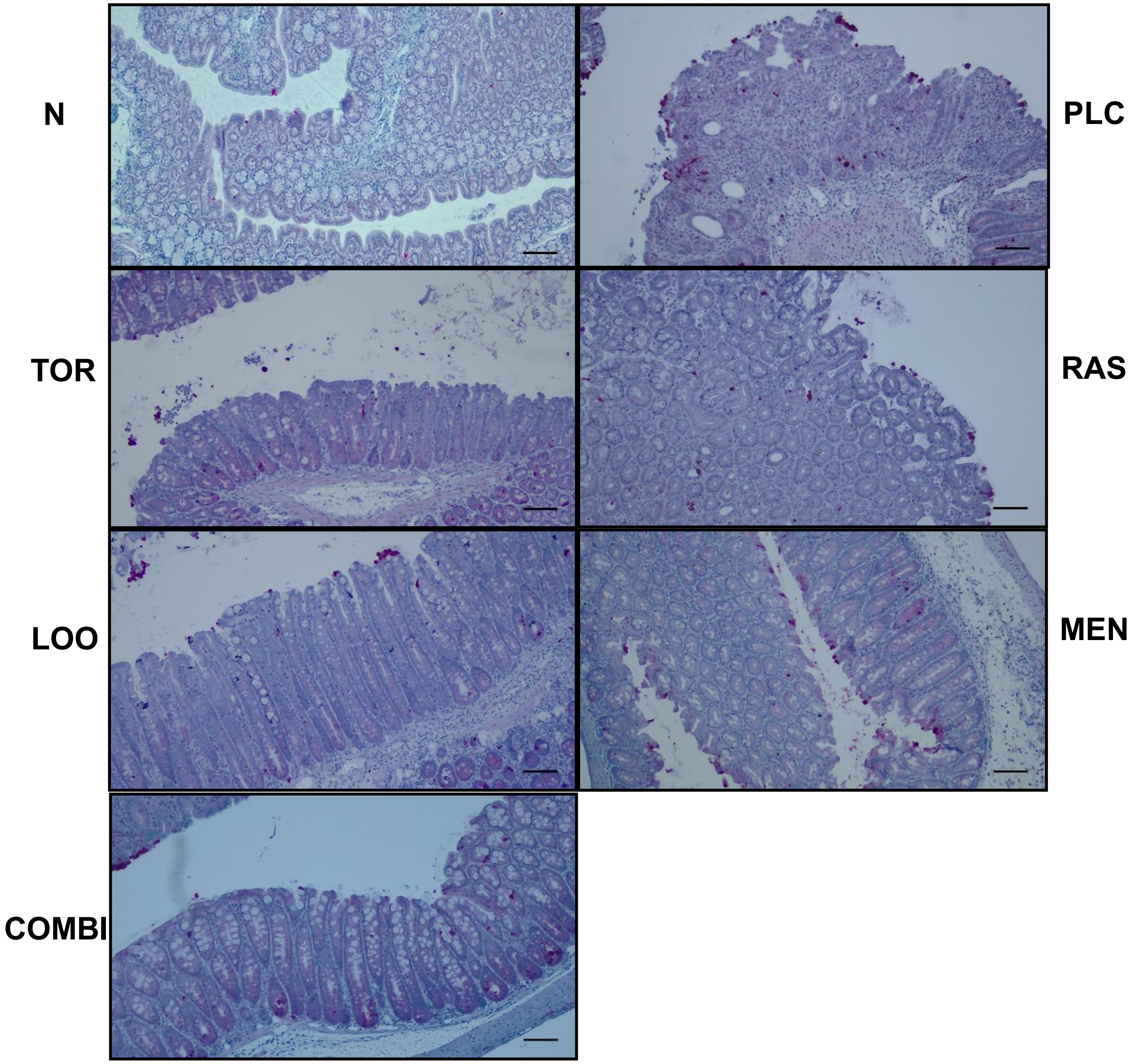

Supplementary Figure S3. Representative photomicrographs illustrating microscopic inflammatory changes following oral treatment of infected gut microbiota-depleted IL-10$^{-/-}$ mice with distinct natural compounds alone or in combination. Mice were orally infected with *C. jejuni* 81-176 strain on days 0 and 1. From day 2 until day 6, mice were treated with either tormentil (TOR), raspberry leaves (RAS), loosestrife (LOO), and menthol (MEN) alone or with a combination of all four compounds (COM) via the drinking water. Placebo control mice received vehicle only (PLC), whereas naive mice (N) served as uninfected and untreated controls. Photomicrographs representative of four independent experiments illustrate **(A)** colonic histopathological changes in hematoxylin and eosin (H&E) stained colonic paraffin sections (100x magnification; scale bar 100 µm) and **(B)** apoptotic colonic epithelial cells in large intestinal paraffin sections positive for cleaved caspase-3 (100x magnification, scale bar 100 µm) on day 6 post-infection.

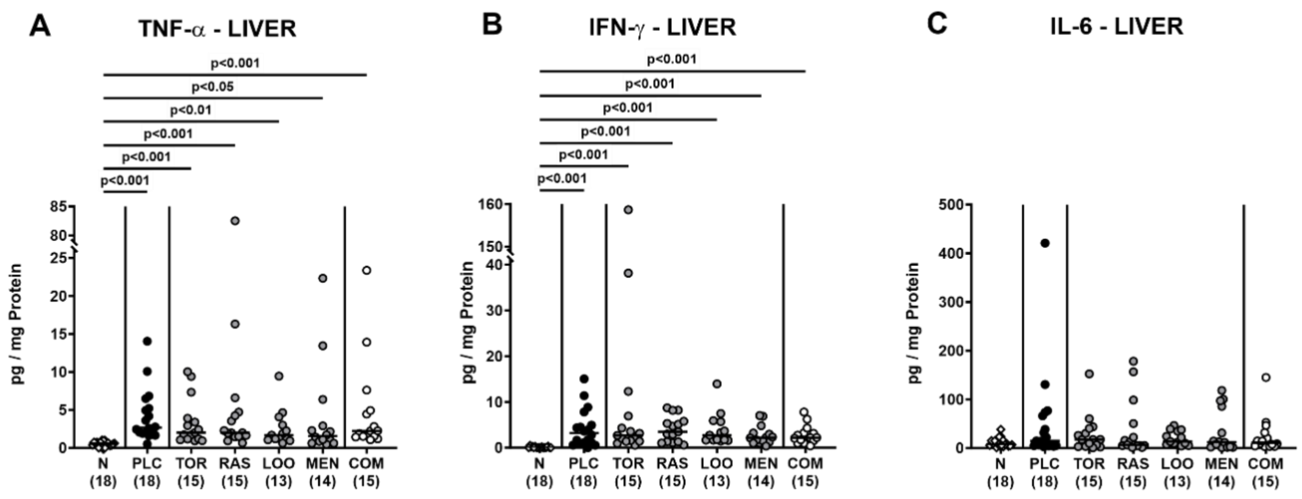

Supplementary Figure S4: Pro-inflammatory cytokine secretion in the liver following oral treatment of infected gut microbiota depleted IL-10$^{-/-}$ mice with distinct natural compounds alone or in combination. Mice were orally infected with *C. jejuni* 81-176 strain on days 0 and 1. From day 2 until day 6, mice were treated with either tormentil (TOR), raspberry leaves (RAS), loosestrife (LOO), and menthol (MEN) alone or with a combination of all four compounds (COM) via the drinking water. Placebo control mice received vehicle only (PLC), whereas naive mice (N) served as uninfected and untreated controls. On day 6 post-infection, **(A)** TNF-α, **(B)** IFN-γ, and **(C)** IL-6 concentrations were measured in liver explants. Medians (black bars), significance levels (p values) determined by the Kruskal-Wallis test with Dunn's multiple comparison test and numbers of mice included from three independent experiments (in parentheses) are indicated.

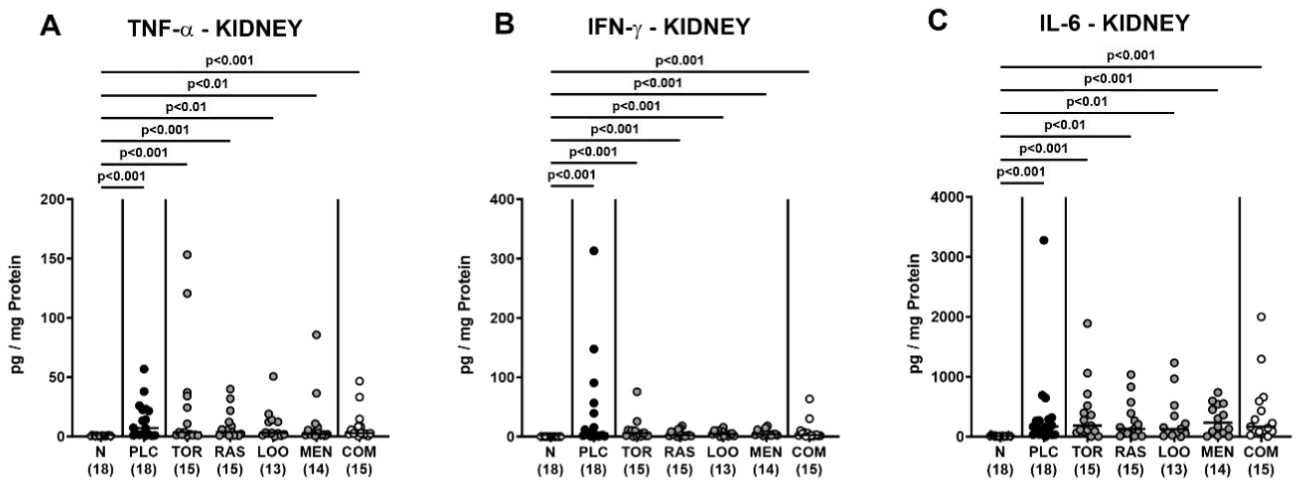

Supplementary Figure S5: Pro-inflammatory cytokine secretion in the kidneys following oral treatment of infected gut microbiota depleted IL-10$^{-/-}$ mice with distinct natural compounds alone or in combination. Mice were orally infected with *C. jejuni* 81-176 strain on days 0 and 1. From day 2 until day 6, mice were treated with either tormentil (TOR), raspberry leaves (RAS), loosestrife (LOO), and menthol (MEN) alone or with a combination of all four compounds (COM) via the drinking water. Placebo control mice received vehicle only (PLC), whereas naive mice (N) served as uninfected and untreated controls. On day 6 post-infection, **(A)** TNF-α, **(B)** IFN-γ, and **(C)** IL-6 concentrations were measured in kidney explants. Medians (black bars), significance levels (p values) determined by the Kruskal-Wallis test with Dunn's multiple comparison test and numbers of mice included from three independent experiments (in parentheses) are indicated.

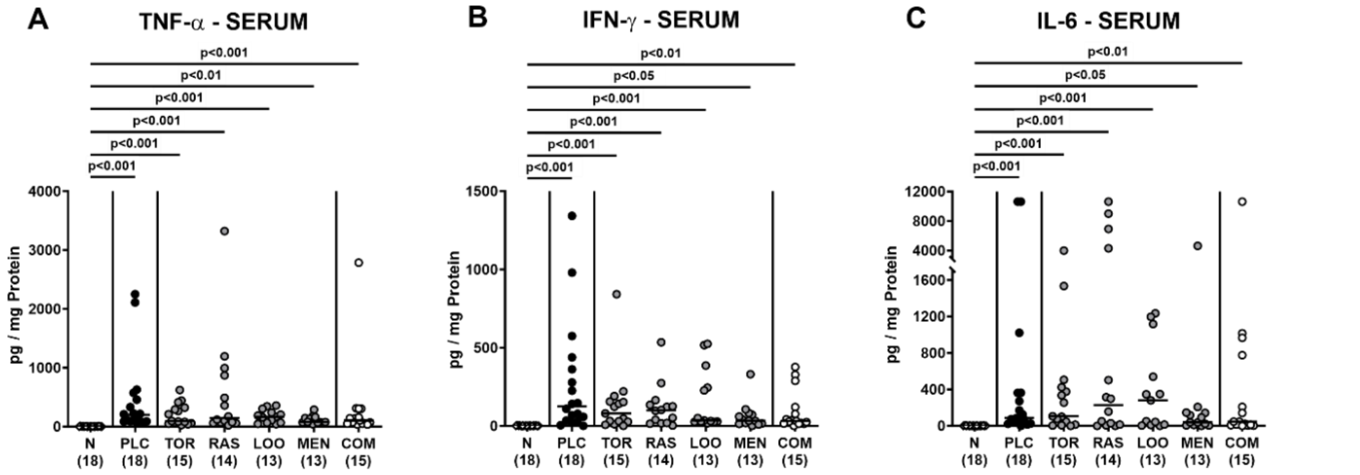

Supplementary Figure S6: Systemic pro-inflammatory cytokine secretion following oral treatment of infected gut microbiota depleted IL-10$^{-/-}$ mice with distinct natural compounds alone or in combination. Mice were orally infected with *C. jejuni* 81-176 strain on days 0 and 1. From day 2 until day 6, mice were treated with either tormentil (TOR), raspberry leaves (RAS), loosestrife (LOO), and menthol (MEN) alone or with a combination of all four compounds (COM) via the drinking water. Placebo control mice received vehicle only (PLC), whereas naive mice (N) served as uninfected and untreated controls. On day 6 post-infection, **(A)** TNF-α, **(B)** IFN-γ, and **(C)** IL-6 concentrations were measured in serum samples. Medians (black bars), significance levels (p values) determined by the Kruskal-Wallis test with Dunn's multiple comparison test, and numbers of mice included from three independent experiments (in parentheses) are indicated.
